# Assessing fall armyworm (*Spodoptera frugiperda*) allochronic behavior as a predictor of local strain composition in United States populations

**DOI:** 10.3389/fpls.2024.1380624

**Published:** 2024-10-24

**Authors:** Andie C. Miller, Ashley E. Tessnow, Robert L. Meagher, Rodney N. Nagoshi, Todd M. Gilligan, Gregory A. Sword

**Affiliations:** ^1^ Department of Entomology, Texas A&M University, College Station, TX, United States; ^2^ Ecology and Evolutionary Biology Interdisciplinary Program, Texas A&M University, College Station, TX, United States; ^3^ Center for Medical, Agricultural, and Veterinary Entomology, United States Department of Agriculture - Agricultural Research Service (USDA-ARS), Gainesville, FL, United States; ^4^ Department of Agricultural Biology, Colorado State University, Fort Collins, CO, United States

**Keywords:** fall armyworm, allochronic, behavioral isolation, temporal isolation, strain-specific monitoring

## Abstract

The fall armyworm, *Spodoptera frugiperda* (J.E. Smith), is comprised of two genetically distinct strains that are morphologically identical yet exhibit differences in their behavior and physiology (C-strain and R-strain). Evidence of ongoing genetic differentiation between strains highlights the importance of considering strain identity in research and management of fall armyworm populations, but the logistical and technical burden of genotyping limits strain-specific applications. Controlled experiments with laboratory colonies have shown that the strains engage in allochronic (“allo” – different, “chronic” – time) mating behavior, with C-strain mating early in the evening (0–5 hours after sunset) and R-strain mating late in the evening (5–10 hours after sunset). Using temporal field collections and genotype data, we show that strain-specific variation in allochronic male mating behavior occurs across Texas and Florida fall armyworm populations, both of which act as primary source populations for annual migrations of this pest into the continental United States. Time of capture in pheromone traps was significantly different between strains in both Texas and Florida, with the R-strain males consistently being collected in the traps late in the night. The C-strain males were generally captured earlier in the night than their R-strain counterparts, though there was notable variation in the timing between nights and across locations. Allochronic behavior in field populations is consistent with previous laboratory studies reporting differences in the timing of mating between the strains, however increased variability in behavior within and across native populations was observed. Although allochronic behavior in local populations may partially contribute to reproductive isolation between the strains, the behavior is not consistent enough to serve as a complete reproductive barrier. Furthermore, the observed variability in behavior both within and between independent sampling events, especially in the C-strain, poses a challenge to the development of models that utilize time of capture as a predictive phenotype for monitoring strain identity in local populations.

## 1 Introduction

The fall armyworm, *Spodoptera frugiperda* (J.E. Smith), is a noctuid moth that is a widespread and highly polyphagous pest of maize, sorghum, cotton, peanut, pasture, turf grasses and many other crops (Pashley, 1988b; Montezano et al., 2018). The species is comprised of two morphologically identical yet genetically distinct strains: the C-strain and the R-strain (Pashley, 1986). Assessments of population structure suggest that the strains exhibit some host association, with C-strain populations associated with large blade grasses, such as corn and sorghum, whereas R-strain populations are more generalist and regularly recovered in corn and sorghum along with turf, pasture, and Bermuda grasses (Pashley, 1988b; Pashley et al., 1995; Meagher and Nagoshi, 2004; Nagoshi and Meagher, 2004a; Nagoshi et al., 2007; Juárez et al., 2012; Murúa et al., 2015).

Knowledge of strain identity in local populations could be beneficial for effective fall armyworm management, as the threat of larval damage to different crop systems varies with strain. Importantly, comparative studies have revealed that the two strains are differentially susceptible to Bt cry proteins, cypermethrin, and other chemistries commonly used to control the fall armyworm in the United States (Adamczyk et al., 1997; Ríos-Díez and Saldamando-Benjumea, 2011; Ríos-Díez et al., 2012). To date, the only accurate and reliable means of strain identification involves expensive and laborious genotyping techniques, the implementation of which limits the feasibility of timely strain-specific management efforts within the United States (Pashley, 1986; Lu et al., 1992; McMichael and Prowell, 1999; Nagoshi et al., 2006a; Nagoshi, 2010; Tessnow et al., 2021). There is growing evidence that the fall armyworm strains are in the intermediate stages of speciation, and continued divergence between the strains could have implications for research and management in the future (Prowell et al., 2004; Reviewed in Nagoshi and Meagher, 2022). Investigating fall armyworm strain ecology and behavior provides the opportunity to better characterize mechanisms that limit their introgression and may permit the identification of strain-specific phenotypes that could serve as diagnostic traits to enhance monitoring and management efforts.

The existence of sympatric, genetically distinct strain populations indicates that barriers to gene flow exist that limit inter-strain hybridization. However, the reproductive isolation mechanism(s) responsible for strain differentiation are not fully understood (Reviewed in Groot et al., 2010). In their endemic range in the Western Hemisphere, the two fall armyworm strains are often considered host strains, as numerous reports indicate statistically significant correlations between host plants and strain-specific genetic markers (Pashley, 1988a; Nagoshi et al., 2007; Juárez et al., 2012; Murúa et al., 2015; Nagoshi, 2022). However, mismatches between strain identity and host plant utilization occur regularly in the field (Prowell et al., 2004; Juárez et al., 2014; Murúa et al., 2015), and there is limited evidence of strain-specific host plant preference and performance in larval feeding assays (Pashley, 1988a; Pashley et al., 1995; Meagher et al., 2004). Nevertheless, host plant association is hypothesized as the predominant mechanism underlying strain diversity despite being an incomplete isolation barrier that still permits inter-strain hybridization, particularly in corn and sorghum habitats (Fiteni et al., 2022; Nagoshi, 2022).

Allochronic (“allo” – different, “chronic” – time) mating behavior is a trait unique to fall armyworm populations in the United States that may further contribute to the maintenance of strain diversity in the field. Multiple laboratory assays indicate that the two different strains mate during distinct portions of the scotophase (i.e., overnight), with the C-strain preferentially calling, mating, and ovipositing earlier in the evening, typically 0–5 hours after sunset (Pashley et al., 1992; Schöfl et al., 2009; Hänniger et al., 2017). R-strain individuals engage in the same mating behaviors; however, they delay mating activities until later in the evening, usually 5–10 hours after sunset. Potentially related to this behavior are heritable polymorphisms in the circadian gene *vrille* that may differ between strains and influence their circadian clocks (Hänniger et al., 2017).

Reports of allochronic mating behavior are limited to studies of fall armyworm populations in the United States, as investigations into Colombian and West African populations failed to identify strain-specific mating periods (Velásquez-Vélez et al., 2011; Saldamando-Benjumea et al., 2014; Hänniger et al., 2020). United States source populations have been broadly categorized based on their migration pathways, with the Texas (“Central flyway”) population consisting of moths that overwinter in subtropical southern Texas and northern Mexico, and act as the source of annual migrants that spread west and north of the Appalachian Mountain Range (Nagoshi et al., 2008, 2009; Westbrook et al., 2016, and Nagoshi et al., 2012; Tessnow et al., 2023). The Florida (“Eastern flyway”) population consists of moths that overwinter in southern Florida and serve as the source of migrants that seasonally spread along the Atlantic coast (Nagoshi et al., 2008, 2009; Westbrook et al., 2016, and Nagoshi et al., 2012). Although strain-specific allochronic mating behavior has been demonstrated in laboratory studies using colonies sourced from Florida and Louisiana (Pashley et al., 1992; Schöfl et al., 2009; Hänniger et al., 2017), more extensive evaluation of these behaviors in the field are required to determine whether strain-specific variation in mating behaviors can be used as a diagnostic trait to monitor local strain identity. Previous reports indicate that variation in fall armyworm colony age and geographical origin may contribute to discrepancies in some strain-specific phenotypes, including egg mass production and viability (Quisenberry, 1991); thus, it is possible that mating behaviors of laboratory colonies are not entirely representative of field populations. Laboratory studies also do not consider other ecological variables that may influence the expression of allochronic behavior in field conditions (e.g., weather, seasonality, etc.). With respect to the expression of allochronic behavior in the field, Tessnow et al. (2022) provided the only report to date of allochronic behavior in the field, and was limited to populations in Texas, USA.

In this study, we used temporally restricted manual captures and time-stamped automated captures of males in pheromone traps to investigate the prevalence and consistency of strain-specific mating behavior across the geographically distinct source populations of fall armyworm in the United States. Collections were conducted in Texas and Florida in the summer and fall to account for geographic and seasonal fluctuations in strain composition (Pashley, 1988b; Pashley et al., 1992; Nagoshi and Meagher, 2004b). Molecular genotyping was used to determine the strain identity of samples captured during known portions of the evening.

## 2 Methods

### 2.1 Sampling locations

Fall armyworm were sampled at locations in Texas and Florida which serve as primary source populations for annual migrations in the continental United States (Westbrook et al., 2016). Trapping was conducted in agricultural plots at the Texas A&M Field Laboratory in Snook, TX (30.5495, −96.4367), the University of Florida Everglades Research and Education Center (EREC) in Belle Glade, FL (26.6532, −80.6389) and surrounding commercial agricultural fields in Palm Beach county, and the University of Florida Tropical Research and Education Center in Homestead, FL (25.50887, −80.50133). Nine moths were collected from the Homestead, FL location. They were identical to the Belle Glade, FL samples in strain identity and temporal distribution, so were pooled with the Belle Glade, FL samples for simplified analysis and reporting. The Texas traps were placed adjacent to corn and sorghum fields with cotton, sunflower, pasture grasses and weeds growing nearby. Florida traps at EREC were similarly placed near corn and sorghum fields with sugarcane and weeds growing nearby. Traps from Florida collections in July 2023 were placed along a farm road in between a commercial sod farm and a commercial rice field. Information on the location, date, and strain composition of fall armyworm male captures can be found in **Supplementary Table 1**.

### 2.2 Manual trap captures

Manual trap captures were conducted using standard Universal moth traps (“bucket” traps with green tops, yellow funnels, and white buckets) (distributed by Great Lakes IPM, Vestaburg, MI, USA). Traps were baited with one of two different pheromone lures, a three-component lure manufactured by Trécé Inc. (Adair, OK, USA) or a two-component lure manufactured by Scentry Biologicals, Inc. (‘PSU’ lure, Billings, MT, USA). Moths were killed in the buckets with the use of insecticide strips (Vaportape II, Hercon Environmental, Emigsville, PA, USA). Previous research showed that in general, both lures attracted similar numbers of moths (Meagher et al., 2013), and that commercial multicomponent pheromone lures regularly capture both C-strain and R-strain males and do not exhibit strain specific biases (Unbehend et al., 2014). Bucket traps were placed >30m apart. Manual bucket trap captures were conducted only in Florida and were not employed in Texas collections. Regardless of the seasonal photoperiod, early and late captures were designated as occurring either before or after solar midnight (i.e., the exact middle of the scotophase). As an example, during a 12L:12D photoperiod, all moths captured during the “early” portion of the evening were physically recovered 6 hours after sunset from a trap that had been set up earlier in the day, while moths captured during the “late” portion of the evening were recovered the next morning from a trap set up 6 hours after sunset. Upon collection, moths were stored at −20°C and preserved in either >95% alcohol or stored dry prior to DNA extraction. Information regarding the frequency of trapping nights (events), strain composition, and the proportion of early and late captures from each sample location can be found in **Supplementary Table 1**.

### 2.3 Automated trap captures

Automated lepidopteran pheromone traps were commercially obtained from Trapview North American LLC (Vancouver, WA, USA). These digital image-based automated traps are modular and equipped with the trapping apparatus, a 12V battery power system, solar panel, digital camera, and mobile phone capability. The trapping apparatus contains the funnel and pheromone lure component of a traditional all green Universal moth trap. As moths enter the trap, they are funneled directly into a collection chamber fitted with a roll of sticky paper at the base upon which the moths become affixed. The sticky paper was photographed at the beginning of every hour from above by a digital camera housed within the collection chamber every night throughout the experiment. Hourly image data from each trap was then uploaded to the Trapview website where it could be checked directly using either a website interface or a mobile phone app. The image data was used to assign a timestamp to each moth that corresponded to the hourly interval of its capture and its position on the sticky paper When the surface of the sticky paper became saturated with moths, the roll could be automatically advanced to provide a clean capture surface.

All five automated traps were baited with a 4-component pheromone lure (Scentry 4, Scentry Biologicals Inc., distributed by Great Lakes IPM). A Vaportape II (Hercon Environmental, distributed by Great Lakes IPM) strip was placed inside each trap to reduce the movement of moths on the sticky paper. Moths were collected from the trap sticky paper 1–3 days after capture, with individuals identified and timestamped using the hourly image data. Samples were preserved in 95% ethanol and stored at −20°C prior to DNA extraction. On evenings when multiple traps were set up at a given location, data from all traps were pooled as a single sampling event (**Supplementary Table 1**).

### 2.4 DNA extraction

Prior to DNA extraction, the thorax of each moth was isolated, surface sterilized using 95% ethanol, and placed in 2-mL cryotubes with two sterile steel beads. Cryotubes were submerged in liquid nitrogen for 30 seconds and immediately placed in a Bullet Blender Tissue Homogenizer (Next Advance, Inc, Troy, NY, USA), which pulverized the samples into a fine powder over the course of 30 seconds. DNA was extracted using the QIAGEN DNEasy Blood and Tissue kit per the manufacturer’s protocol (QIAGEN Sciences, Germantown, MD, USA). The concentration of each DNA sample was quantified using the Qubit dsDNA Broad Range Quantification Assay (Thermo Fisher Scientific, Waltham, MA, USA), and each sample was diluted to a concentration of 10-20 ng/μL with TE buffer.

### 2.5 Sample strain determination

Nights in which less than 30 moths were collected were prioritized for genotyping to increase the number of independent sampling events that were assessed for strain composition. However, for nights when more than 30 moths were captured, subsampling was required. When subsampling was conducted, an effort was made to genotype the same number of moths captured early and late in the evening to avoid biasing the dataset.

The strain identity of collected moths was determined using the diagnostic TaqMan assays described in Tessnow et al. (2021). Briefly, for all samples, real-time PCR assays were conducted as 10 μL reactions in 384 well plates. Each reaction was comprised of 1μL of diluted template DNA (described in section 2.4), 5 μL TaqMan Genotyping Master Mix (Thermo Fisher Scientific, Waltham, MA, USA), 0.5 μL of 40x Custom Thermo Fisher TaqMan assay containing the primers and hydrolysis probes, and 3.5 μL of TE buffer (Tessnow et al., 2021). TaqMan assay “A”, “C”, and “D” were identified and described by Tessnow et al. (2021) and were used in this assay to determine the strain of each sample. The SNPs leveraged by TaqMan assay “A” and “C” are located on the Z- chromosome, while TaqMan assay “D” utilizes a SNP located on an autosome. All three TaqMan assays can identify homozygous and heterozygous males, meaning strain identity was determined utilizing three distinct diagnostic regions in the genome. Non-template controls included 1 μL of TE buffer rather than DNA and positive controls were generated from pure C-strain and R-strain colonies that are maintained at Texas A&M University and regularly checked for strain purity. All assays were run in duplicate on a CFX384 Touch Real-Time PCR Detection System (Bio-Rad, Hercules, CA, USA) as described by Tessnow et al. (2021). The real-time PCR protocol began with samples held at 95°C for 10 minutes, followed by 40 cycles oscillating between 95°C for 15s to 60°C for 1 min. After being held at 60°C for 1 minute, fluorescence was recorded across all four channels of the real-time PCR machine and real-time PCR data were assessed using the CFX Maestro software (Bio-Rad, Hercules, CA, USA) for strain identification.

The CFX Maestro software identifies samples as either homozygous for the R-strain allele (primarily FAM fluorophore detected), homozygous for the C-strain allele (primarily VIC fluorophore detected), or heterozygous at the locus of interest (both FAM and VIC fluorophores detected at similar levels). Samples with majority VIC fluorophores at SNP “A”, “C”, and “D” were identified as C-strain. Samples with majority FAM fluorophores at SNP “A” and “C” and heterozygote at SNP “D” were identified as R-strain. In previous studies, TaqMan assay “D” has indicated binding of both target and non-target probes to the DNA sequence containing SNP D, suggesting a lack of probe specificity (Tessnow et al., 2021). As a result, individuals that are homozygous for the R-strain allele are often called heterozygous using the SNP D marker. All suspected C-strain and R-strain samples were compared to positive controls to confirm strain identity. Samples that were identified as heterozygous at SNP “A” or SNP “C” were considered putative hybrids and were excluded from further analysis. Samples with incongruent strain calls (ex: VIC, FAM, VIC at SNP A, C, and D) were also considered putative hybrids and were excluded from further analysis. A total of 362 samples were genotyped, of which 176 (48.6%) were identified as C-strain, 154 (42.5%) were identified as R-strain, and 32 (8.8%) were identified as putative hybrids (**Supplementary Table 1**).

### 2.6 Statistical analysis of temporal differences in strain activity in the field

General linearized models were used to test the effect of location and time on the probability of collecting C-strain and R-strain moths as determined by genotyping in a combined dataset of both the manual and automated trap captures. To maintain consistency between automated and manual trap time intervals, moths were classified as “early” if they were collected before solar midnight, and “late” if they were collected after solar midnight. The hourly nature of image data from the automated traps hindered the ability to utilize exact solar midnight for early and late classifications. For example, sunset in Texas in June of 2023 was approximately 20:30, meaning solar midnight was 1:30. Image data from automated traps can only identify moths collected from 1:00 – 2:00. Therefore, all moths collected between 1:00 – 2:00 were considered early and the “early” and “late” classifications were adjusted to ensure that each collection period was the same duration. Collections from automated traps in June and July 2023 in Texas categorized early moths as those captured between 0 – 5.5 hours after sunset and late moths as those captured 5.5 hours after sunset to sunrise. For collections in automated traps in Texas in October – November 2023, early moths were collected 0 – 6.5 hours after sunset and late moths were collected 6.5 hours after sunset to sunrise. Automated collections in Florida in September 2023 contained early moths collected 0 – 6.5 hours after sunset and late moths 6.5 hours after sunset to sunrise. Manual trap captures allowed for moths to be collected more precisely at solar midnight, therefore for collections in April 2022 early moths were collected 0 – 6 hours after sunset and late moths were collected 6 hours after sunset to sunrise. Manual captures in July 2023 contained early moths collected 0 – 5 hours after sunset and late moths captured 5 hours after sunset to sunrise. Information regarding the location, date, strain composition, and temporal frequency of captures can be found in **Supplementary Table 1**. To account for variation in the strain composition of collections from Texas and Florida populations, the effect of location was considered in all analyses of time as a predictor of strain identity. Limited R-strain abundances in Texas from June – July 2023 and C-strain abundances in Florida in July and September 2023 prohibited assessments of seasonal effects on time of capture. Generalized linear models with binomial distributions and a logit link were conducted in JMP v.16 (SAS Institute Inc. Cary, NC, USA).

In total, 42 independent sampling events (i.e., total moths collected in one evening pooled across traps at a given location) were conducted in Texas in 2023 and Florida from April 2022 – November 2023; 5 events using manual traps in Florida and 37 events using automated traps across both locations. Individual moths collected in automated traps were associated with a specific hourly time of capture. To determine if the median time of capture of individuals from the automated traps was significantly different between strains for males collected in Texas and Florida, Mann Whitney U Tests were conducted in JMP v.16. The effect of location was tested through within-strain comparisons of median rank time for samples collected in Texas and Florida. The lower temporal resolution of moths collected in manual traps, which provide binary “early” and “late” classifications, prohibited their inclusion in the analysis.

Given that comparing median capture times based on individuals pooled from the entire data set could be biased by larger collections on specific nights, we also calculated the median capture time per independent sampling event (i.e., total moths collected in one evening at a given location) and compared these values between strains and locations. Sampling events with less than three moths collected were excluded from median calculations, with a total of 196 moths from 19 independent sampling events analyzed.

To assess the consistency of strain-specific allochronic behavior across sampling events, binomial exact tests were conducted to test whether the frequency of independent sampling events in which 50% or more of the total number of C-strain males captured and genotyped were collected before solar midnight differed from that expected by chance alone. Likewise, the same test was conducted for R-strain males assessing the frequency of events in which 50% or more of the total number of R-strain males captured were collected after solar midnight. The analysis included males collected in both manual and automated traps during sampling events in which three or more moths were collected for a total of 24 nights assessed. Six separate binomial exact tests were conducted to assess variation in nightly C-strain and R-strain time of capture in Texas only, Florida only, and both locations combined. Binomial exact tests with a two-sided chi-square test for the alternative hypothesis were conducted in JMP v.16 (SAS Institute Inc. Cary, NC, USA).

To determine if time of capture could be used as a predictor of local strain composition based on independent sampling events, the linear correlation between the proportion of early relative to total trap captures and the proportion of C-strain genotypes from the same sampling event was quantified across all sampling events using Pearson’s correlation coefficient. The same correlation can also be calculated using the proportion of late captures and the proportion of R-strain genotypes for each sampling event but is mathematically equivalent to the C-strain analysis. Samples from manual and automated trap captures were assigned “early” and “late” classification as was previously described for analysis via general linearized models. Assessments of the correlation between the proportion of early and late captures and the proportion of C-strain and R-strain captures were conducted using sampling events in which three or more moths were collected in all locations (N = 24), Texas sampling events only (N = 15), and Florida sampling events only (N = 9). Correlations were conducted in JMP v.16 (SAS Institute Inc. Cary, NC, USA).

## 3 Results

**Figure 1:**
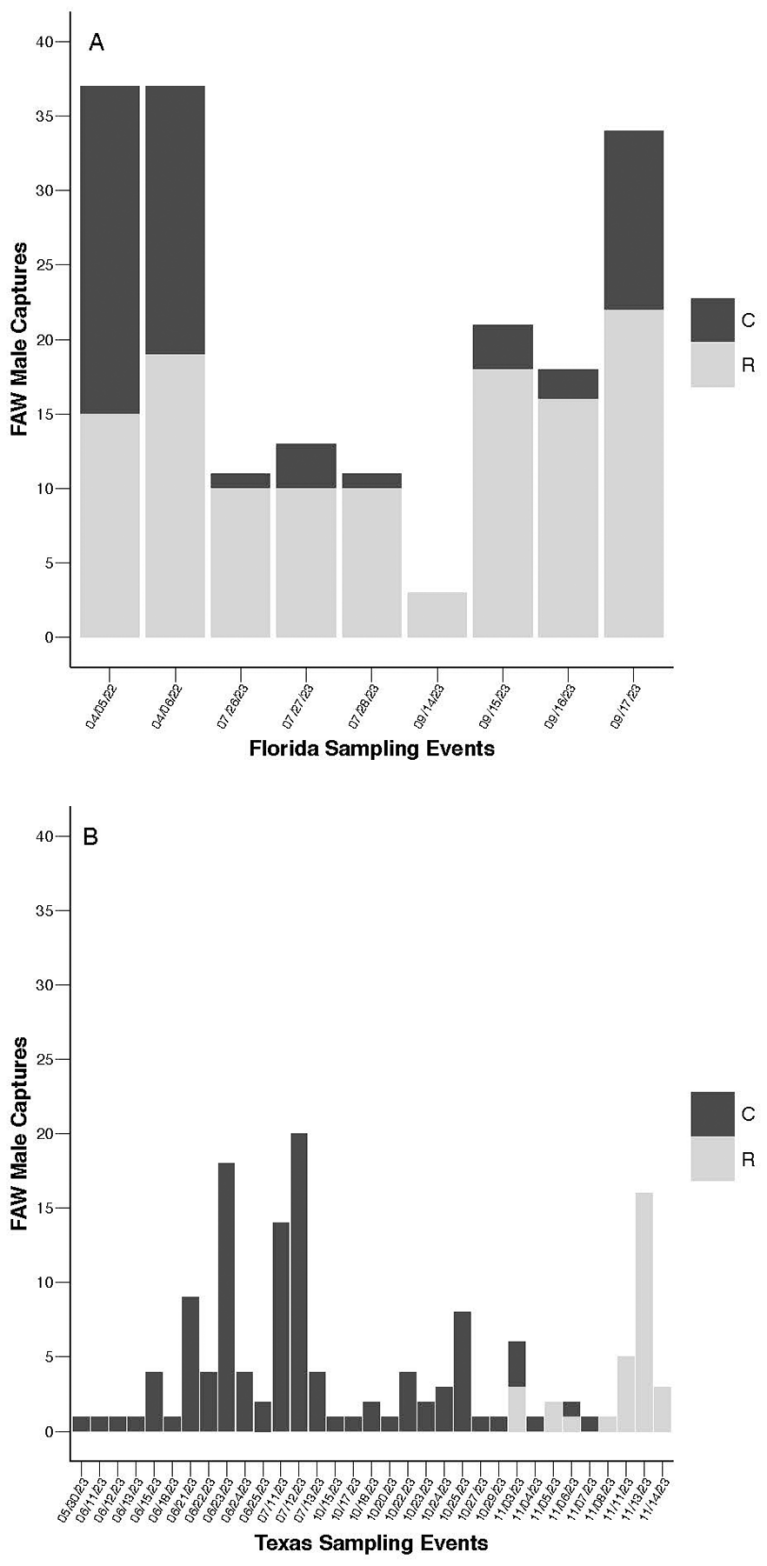
Total fall armyworm (FAW) captures per independent sampling event. **(A)** Frequency of C-strain (black) and R-strain (grey) male captures across sampling events in Florida in 2022 – 2023 (N_C_ = 62, N_R_ = 123). **(B)** Frequency of C-strain (black) and R-strain (grey) male captures across sampling events in Texas 2023 (N_C_ = 114, N_R_ = 31). Analysis using generalized linear models reveals that location had a significant effect on the probability of collecting R-strain males (N = 154, ***x* ^2^** = 28.375, df = 1, P < 0.0001), but not C-strain males (N = 176, ***x* ^2^** = 2.470, df = 1, P = 0.1161).

The strain compositions of field populations in both Texas and Florida were unbalanced, with Texas collections biased toward C-strain captures (C = 114, R = 31) and Florida collections dominated by R-strain captures (C = 62, R = 123) (**Figure 1**; **Supplementary Table 1**). Within strain comparisons across Texas and Florida datasets reveal that location had a significant effect on the probability of capturing R-strain males (N = 154, ***x* ^2^** = 28.375, df = 1, P < 0.0001), but not C-strain males (N = 176, ***x* ^2^ =** 2.470, df = 1, P = 0.1161). To account for the effect of location on R-strain captures, Texas and Florida datasets were analyzed independently in subsequent analyses as appropriate.

### 3.1 Analysis of time of capture as a predictor of individual strain identity

Strain-specific differences in the time of capture of C-strain and R-strain males were broadly consistent across Texas and Florida populations. General linearized models were conducted on individual sample data to determine if time of collection before or after solar midnight is a significant predictor of strain identity. In Texas, time had a significant effect on the probability of catching C-strain and R-strain males (N = 145, *x* ^2^ = 5.2670, df = 1, P = 0.0217). C-strain captures were biased toward the early portion of the evening relative to R-strain captures. C-strain moths comprised 87% of early captures, but since they were much more numerically abundant, they also comprised 70% of late captures (**Supplementary Table 1**). R-strain captures represented only 13% of early captures and were collected relatively more often late as 30% of the total late captures (**Supplementary Table 1**). The difference in timing between strains was more robust in Florida populations, with early captures comprised of 96% C-strain and 4% R-strain while late captures were comprised of 8% C-strain and 92% R-strain (**Supplementary Table 1**). Time had a significant effect on the probability of catching C-strain and R-strain males in Florida (N = 185, *x* ^2^ = 129.2260, df = 1, P <0.0001). Taken together, these results indicate that broad time of capture classifications (early = before solar midnight; late = after solar midnight) are significant predictors of strain identity for individual males collected in both Texas and Florida.

### 3.2 Analysis of hourly trap entry intervals per individual

**Figure 2:**
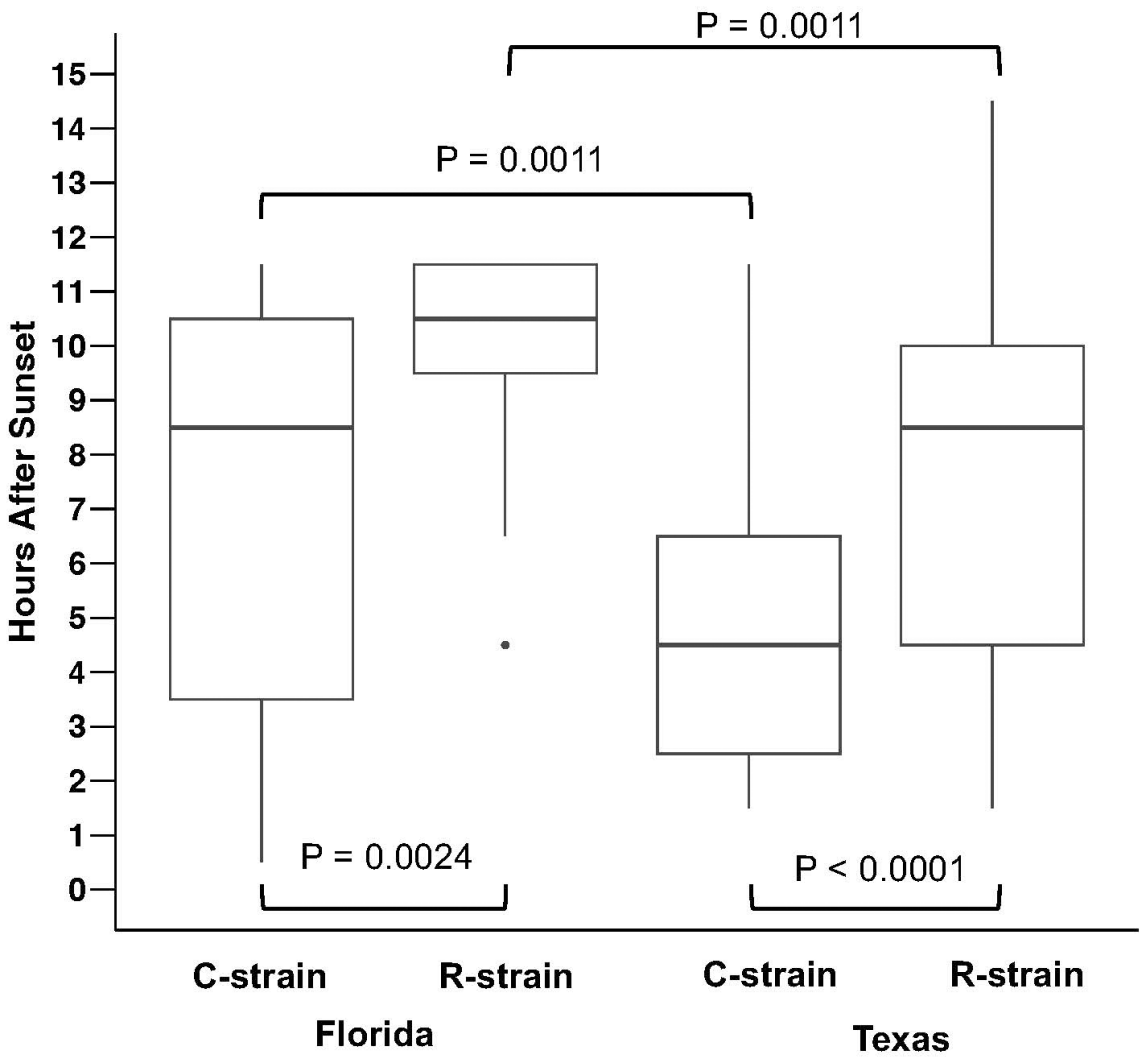
Between strain comparisons of the time of capture of individual male moths using automated trap captures in College Station, TX and Belle Glade, FL. Boxplots show time of capture in hours after sunset (median ± IQR and range) for C-strain and R-strain males. Significant differences were observed between C-strain and R-strain time of capture in Florida (Mann-Whitney U-test, Z = −3.03557, N_C_ = 17, N_R_ = 59, P = 0.0024) and between C-strain and R-strain time of capture in Texas (Mann-Whitney U Test, Z = 3.97211, N_C_ = 114, N_R_ = 31, P< 0.0001). Within-strain comparisons indicate significant differences between the time of capture of C-strain collected in Florida and Texas (Mann-Whitney U Test, Z = 3.27365, N_TX_ = 114, N_FLA_ = 17, P = 0.0011) and R-strain collected in Florida and Texas (Mann Whitney U Test, Z = −3.25444, N_TX_ = 31, N_FLA_ = 59, P = 0.0011).

Differences in the time of capture associated with individual males of each strain were assessed using higher temporal resolution hourly capture data collected with the automated traps. In Florida, C-strain individuals were captured significantly earlier than R-strain (Z = −3.03557, N_C_ = 17, N_R_ = 59, P = 0.0024) with the median capture time for C-strain males approximately 2 hours earlier than R-strain males (**Figure 2**). Texas populations also exhibited significant differences in the time of capture between the strains (Z = 3.97211, N_C_ = 114, N_R_ = 31), P< 0.0001) with a 4 hour difference in the median time of capture of individuals from each strain (**Figure 2**).

Although these findings are consistent with previous reports of allochronic differences between C-strain and R-strain mating behavior, the observed times of capture for each strain exhibited a novel geographic difference. C-strain males from Texas were captured significantly earlier than C-strain males in Florida (Z = 3.27365, N_TX_ = 114, N_FLA_ =17, P = 0.0011) (**Figure 2**). Median time of capture for C-strain individual males collected in Texas and Florida was 4.5 and 8.5 hours after sunset, respectively. R-strain capture times were also significantly different across locations (Z = −3.25444, N_TX_ = 31, N_FLA_ =59, P = 0.0011) with a median time of capture of 8.5 and 10.5 hours after sunset in Texas and Florida, respectively (**Figure 2**).

### 3.3 Analysis of median time of capture per event

**Figure 3:**
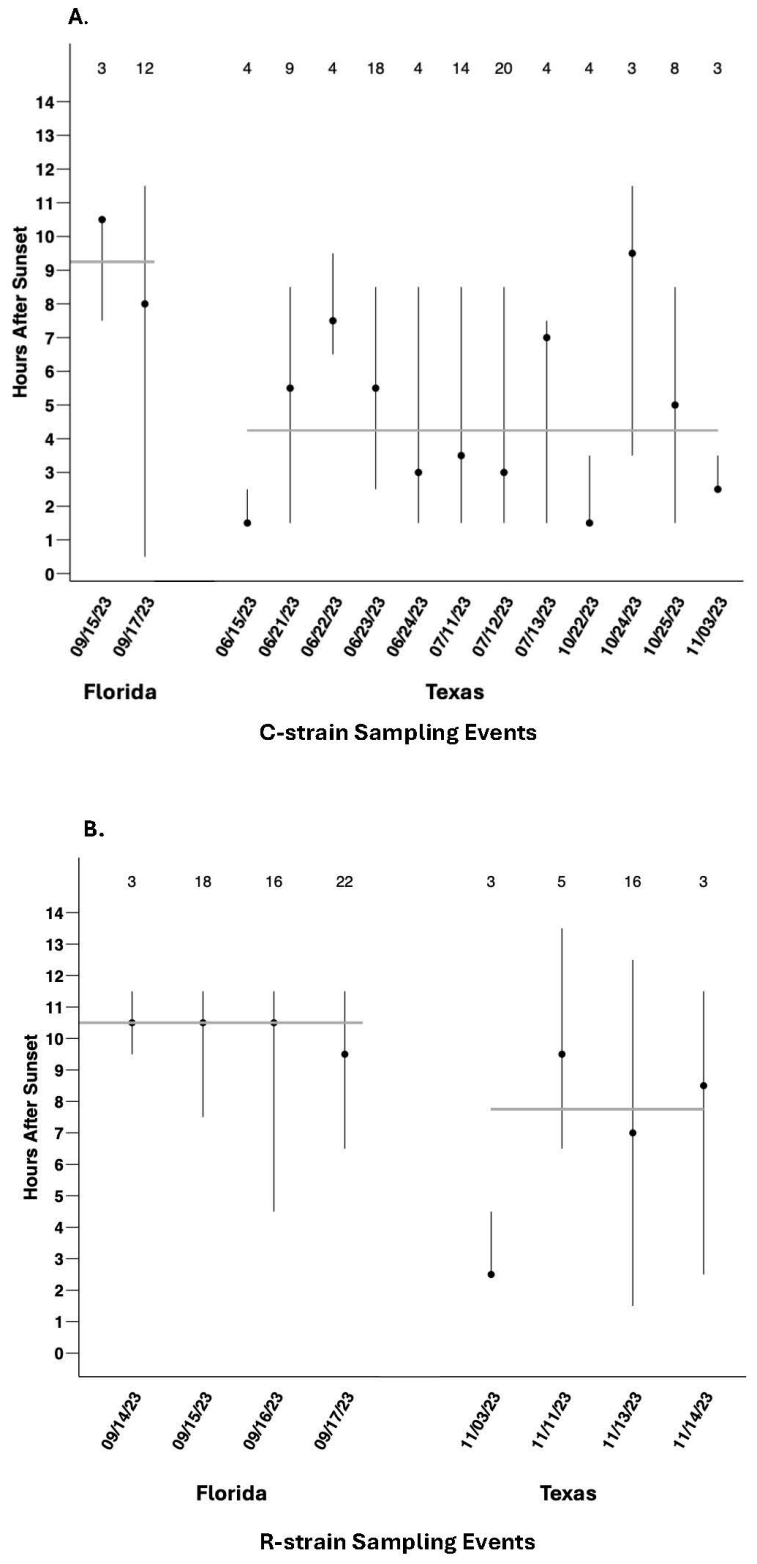
Range and median time of capture of males collected in automated traps during sampling events in which three or more fall armyworm males were captured. **(A)** C-strain males collected during each sampling event in Florida (total N = 15) and Texas 2023 (total N = 95). The C-strain median time of capture across all Florida events is represented by the grey horizontal line at 9.25 hours after sunset. The C-strain median time of capture across all Texas events is represented by the grey horizontal line at 4.25 hours after sunset **(B)** R-strain males collected during each sampling event in Florida (total N = 59) and Texas 2023 (total N = 27). The R-strain median time of capture across all sampling events in Florida is represented by the grey horizontal line at 10.5 hours after sunset. The R-strain median time of capture across all sampling events in Texas is represented by the grey horizontal line at 7.75 hours after sunset. Values above each sampling event represent total C-strain or R-strain male captures used for calculating medians.

Because the number of males collected per sampling event was highly variable for each strain (**Figure 1**), calculation of the median time of capture across all individuals (**Figure 2**) could be biased by the effects of specific events involving large captures and might obscure biologically relevant event-to-event variability. To control for this effect, the median time of capture for C-strain and R-strain males was calculated separately for each independent sampling event in which three or more males were captured (**Figures 3A, B**). Considering the median time of capture per independent sampling event as opposed to the median of all individual capture times did not change the overall pattern of R-strain captures occurring later than C-strain capture regardless of location. The median time of capture of C-strain collections across sampling events in Texas was 4.25 hours after sunset and 9.25 hours after sunset in Florida (**Figure 3A**). The R-strain median time of capture data was more consistent across locations, with a median of 7.75 hours after sunset for collections in Texas compared to 10.5 hours after sunset in Florida (**Figure 3B**).

**Figure 4:**
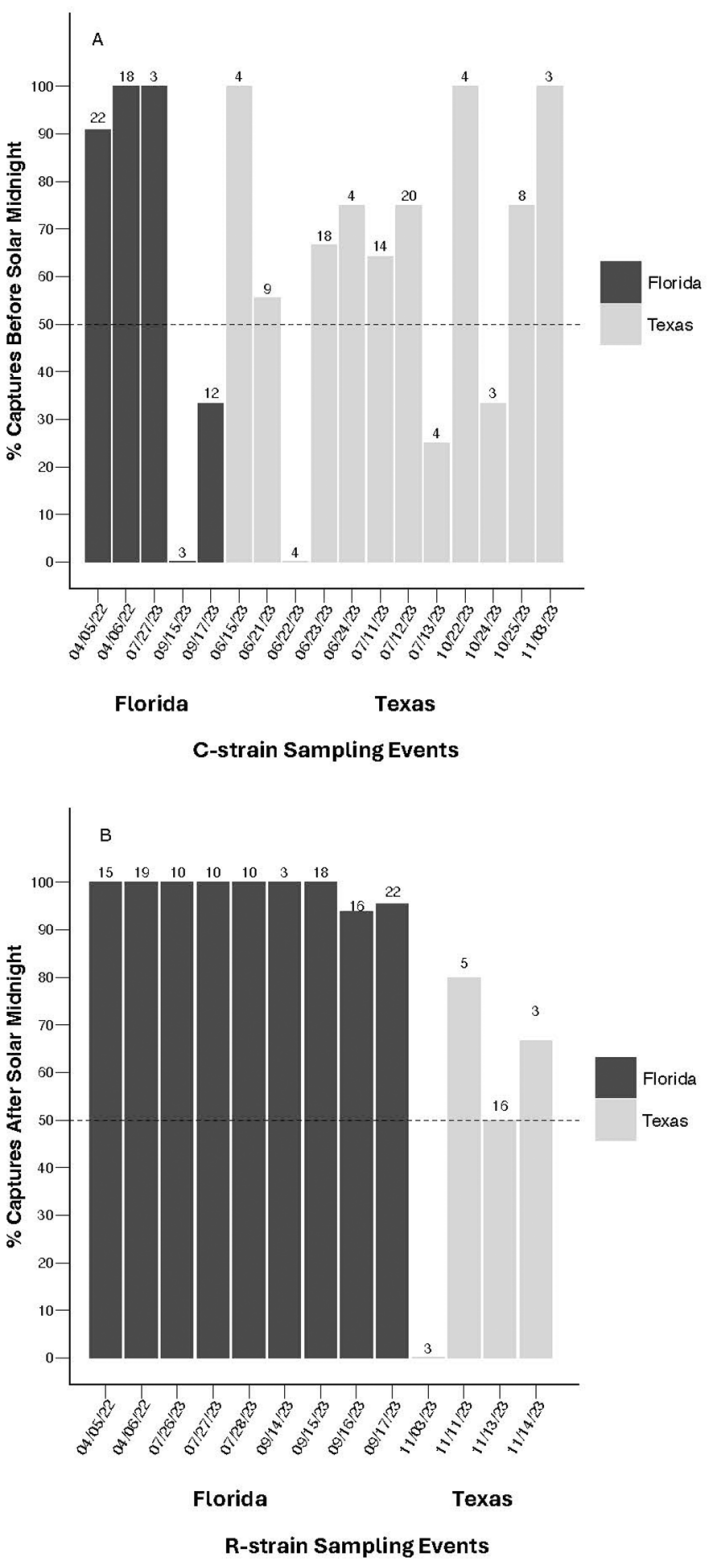
Proportion of fall armyworm males collected either before or after solar midnight from manual and automated traps during sampling events with three or more captures. **(A)** Proportion of C-strain individuals collected before solar midnight from each sampling event using both manual and automated traps in Florida 2022-2023 and from automated traps in Texas 2023. Binomial exact tests indicate that the probability of collecting C-strain males either before or after solar midnight across events does not differ from chance alone in both Florida (N = 5, P = 0.6536) and Texas (N = 12, P = 0.0833). **(B)** Proportion of R-strain individuals collected after solar midnight during each sampling event from manual and automated traps in Florida 2022-2023 and from automated traps in Texas 2023. Binomial exact tests indicates that the probability of collecting R-strain males either before or after solar midnight was significantly different from chance alone across sampling events in Florida (N = 9, P = 0.00195), with catches biased toward after solar midnight. In Texas, the probability of collecting R-strain males either before or after solar midnight was not significantly different from chance alone across sampling events (N = 4, P = 0.3173). Values above each sampling event represent total C-strain or R-strain captures per evening.

To further assess the consistency of allochronic behavior from event to event, binomial exact tests were conducted to determine if C-strain captures across independent events were consistently biased toward before solar midnight. The probability of observing C-strain sampling events in which 50% or more of the total captured C-strain individuals were collected before solar midnight did not differ from chance alone across events in Texas (N = 12, P = 0.0833), Florida (N = 5, P = 0.6536), or when pooled across locations (N = 17, P = 0.0896). This indicates that C-strain captures were not consistently biased toward early evening at the population level on a night-by-night basis, reflecting variability in C-strain time of capture both within and across locations (**Figure 4A**). In contrast, R-strain sampling events were consistently biased toward late evening on a night-by-night basis in Florida (N = 9, P = 0.0020) and across both locations pooled together (N = 13, P = 0.0023) but not in Texas alone (N = 4, P = 0.3173) (**Figure 4B**).

### 3.4 Correlations between time of capture and population strain composition across sampling events

**Figure 5:**
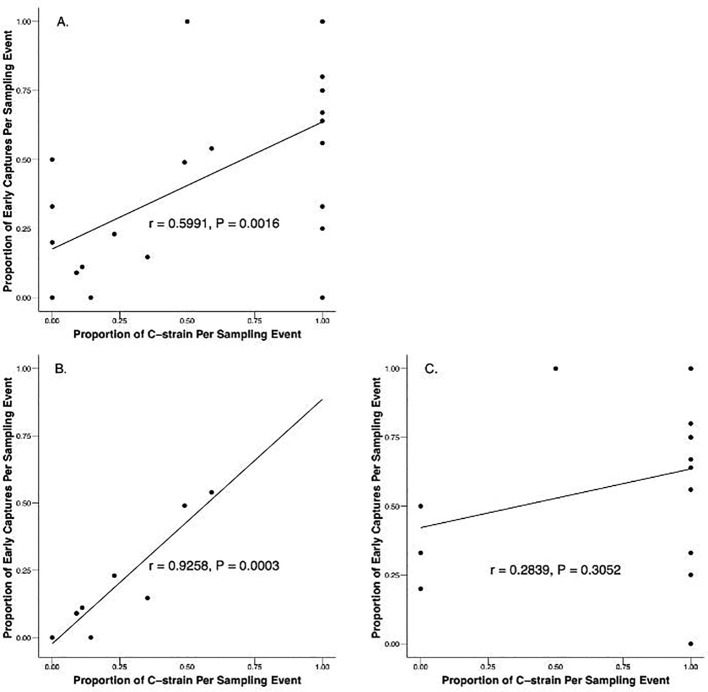
Correlation of the proportion of C-strain and proportion of early captures from manual and automated traps during sampling events in which three or more males were captured. **(A)** All data from Texas and Florida combined (*r* = 0.5991, N = 24, P = 0.0016). **(B)** Florida in 2022 and 2023 (*r* = 0.9258, N = 9, P = 0.0003). **(C)** Texas 2023 (*r* = 0.2839, N = 15, P = 0.3052).

Pearson’s correlation coefficients were calculated to assess the relationship between time of capture and proportional strain composition of local populations to determine if collection time was an effective predictor of strain identity across independent sampling events in which three or more moths were collected. A significant correlation was found between the proportion of C-strain and R-strain in the population as determined by genotyping and the proportion of early and late captures when considering all sampling events together (*r* = 0.5991, N = 24, P = 0.0016)(**Figure 5A**). When assessed by location, a strong, statistically significant association exists between the proportion of early and late captures and the proportion of C-strain and R-strain captures across sampling events in Florida (*r* = 0.9258, N = 9, P = 0.0003) (**Figure 5B**). However, a non-significant relationship exists between the proportion of early and late captures and the proportion of C-strain and R-strain males across sampling events in Texas (*r* = 0.2839, N = 15, P = 0.3052)(**Figure 5C**).

## 4 Discussion

The coexistence and persistence of two genetically distinct fall armyworm strains in field populations implies the presence of intrinsic or extrinsic factors maintaining reproductive isolation. However, hybridization between the strains regularly occurs (Prowell et al., 2004; Nagoshi et al., 2006b; Tessnow et al., 2022) as determined by the mismatch of strain-diagnostic mitochondrial and nuclear genetic markers. Detectable levels of gene flow would not be surprising between the strains if they are at an intermediate stage of speciation as previously proposed (Prowell et al., 2004), with perhaps multiple processes combining to sufficiently limit gene flow and allow strain divergence. We believe this to be a likely scenario that provides an opportunity to evaluate prezygotic mating barriers and develop these as behavioral indicators of the strain composition of local populations. Perhaps the best supported potential barrier is allochronic variation in mating behavior. Allochronic mating behavior was described in laboratory studies as significant strain differences in the timing of female calling behavior and copulation, though with substantial variability in the overlap between strains (Pashley et al., 1992; Schöfl et al., 2009; Hänniger et al., 2017). The likely relevance of these observations to wild populations was indicated by the demonstration that a related field behavior, male attraction to female pheromone in traps, also exhibited strain differences in timing (Tessnow et al., 2022). However, substantial variation in the timing specificity of the C-strain between two Texas locations suggested that complexities in the environment or in the genetic composition of wild populations could significantly compromise the specificity observed under controlled laboratory conditions. To better assess whether allochronic strain mating behavior is a general trait in United States field populations and, if so, whether it is consistent enough to be predictive of strain identity, this field study was extended to compare fall armyworm populations in both Texas and Florida. These regions represent the two major overwintering sources of fall armyworm infestations in North America.

Our data reveal that allochronic differences in behavior between the strains are prevalent and broadly consistent across major geographical source populations. This is true when considering the expression of allochronic behavior across all individuals as well as across independent sampling events. Linear model assessments of individual capture data revealed that broad time classifications (early = before solar midnight; late = after solar midnight) are significant predictors of strain identity for individual moths in both major source populations. These findings are further supported by hourly trap capture data that reveal that the median time of capture of individual C-strain males is significantly earlier in the evening than R-strain males across locations, with at least a two-hour difference in median time of capture between the strains.

Although consistent differences were observed in the median time of capture between strains, these differences appear to be driven by the R-strain males that are consistently attracted to female pheromone late in the scotophase. This trend persists when comparing the behavior of the strains across sampling events in addition to comparing individual behavior. The median time of capture for Texas R-strain sampling events was 7.75 hours after sunset and 10.5 hours after sunset in Florida (**Figure 3B**). Of the 13 sampling events in which more than three R-strain males were captured, 12 events had more than 50% of R-strain captures occur after solar midnight (**Figure 4B**). Our automatic trapping data suggests that C-strain behavior is much less consistent. The median time of capture for C-strain Texas sampling events was 4.25 hours after sunset compared to 9.25 hours after sunset in Florida (**Figure 3A**). Binomial tests reveal that the likelihood of capturing more than 50% of C-strain males in a given trap before solar midnight was not significantly different from chance across multiple independent sampling events (e.g., nights). Additionally, collections in which greater than 50% of C-strain males were captured before solar midnight occurred in only 12 of 17 sampling events (**Figure 4A**). Although a significant, positive correlation exists between the proportion of early and late captures and the proportion of C-strain and R-strain males collected within a given population (**Figure 5A**), this positive relationship is likely influenced by more constrained R-strain behavior. When broken down by location, the correlation between the proportion of early and late captures and the proportion of strains remained significant in Florida but was weaker in Texas. The overrepresentation of C-strain collections in Texas and the greater variation in C-strain time of capture likely resulted in the weaker correlation observed in these sampling events. Together, these observations suggest that the C-strain males are less consistent in their nightly mating times.

In addition to observing the expected strain-specific differences in allochronic behavior that were expressed in both the Texas and Florida populations, we also noted significant differences in the time of capture between the two regions. Both C-strain and R-strain males were captured significantly later after sunset in Florida than those in Texas (**Figures 2**, **3A, B**). However, our manual trap captures from Florida, which provided robust evidence for allochronic strain captures, seemed to collect the C-strain earlier than the automated traps used to determine hourly collection times. Additionally, sampling events using automated traps yielded limited C-strain captures in Florida (N = 17) and R-strain captures in Texas (N=27). As such, although this difference is noteworthy, it should be further investigated to ensure it is not an artifact of low sample sizes.

While this study was not explicitly designed to test for a role of allochronic behavior in fall armyworm strain divergence, the results do suggest that temporal reproductive isolation between strains may be too inconsistent to act as the sole prezygotic barrier to mating. Even if allochronic behavior does act at times as a mechanism for reproductive isolation, it probably functions in consort with other non-mutually exclusive and imperfect barriers to gene flow including host plant association (Fiteni et al., 2022; Nagoshi, 2022). Furthermore, the observed variability in behavior both within and between independent sampling events, especially in the C-strain, suggests that allochronic behavior is too variable to replace genotyping when strain identification must be absolute. Importantly, associations with host plants and allochronic behavior are the only reported phenotypes correlated with strain in North American populations, and therefore remain useful for broader predictions of local strain identity. Host use patterns may convey some degree of information about local strain identity during ongoing larval infestations, while time of capture of adults can provide an early warning of potential strain composition before larval infestations occur. Though not entirely predictive of strain identity in either case, both approaches can nevertheless help inform strain-specific monitoring and forecasting efforts in the United States.

Additional field studies are necessary to assess how well allochronic male captures correlate with the timing of copulation under field conditions, and to determine the influence of biological and ecological factors (e.g., weather, photoperiod, mate competition, migration) on strain mating times. Notably, due to limited sampling events in Florida 2023 and reduced R-strain collections in Texas, it remains unclear if there is consistent geographic variation in the timing of male behavior across populations, especially in the C-strain. In the future, multiyear, season-long assessments of allochronic behavior in field populations should be used to consider the effects of season and photoperiod shifts on the timing of mating behavior. These factors were not assessed in this study but are becoming increasingly tractable with the use of the automated monitoring systems as used here. Additionally, future studies should attempt to assess the time of capture of female moths, as information regarding the timing of their mating activities in the field is entirely absent from the literature. Lab studies suggest that female behavior influences the timing of mating more than male behavior, indicating that studying females could be valuable for the development of behavioral models used for predictive strain identification (Pashley et al., 1992; Schöfl et al., 2009). However, the lack effective trapping technologies for female moths remains a major impediment to such studies in the field.

In summary, our data confirms the strong bias of R-strain males to become more active and receptive to female pheromone after solar midnight and significantly later than their C-strain male conspecifics. This phenotype was observed in both Texas and Florida populations, indicating it is likely to be a general trait exhibited by North American fall armyworm populations. In contrast, equivalent support was not found for biased C-strain male activity prior to solar midnight, as C-strain male captures both at the individual and population level were generally more variable with evidence of seasonal and regional differences. These observations are not entirely consistent with the conclusions of previous laboratory studies, suggesting that the reported strain differences in mating behavior are probably more nuanced and variable in wild populations.

## Data Availability

The original contributions presented in the study are included in the article/**Supplementary Materials**, further inquiries can be directed to the corresponding author.
