# Supplementary material for: Assessing fall armyworm (Spodoptera frugiperda) allochronic behavior as a predictor of local strain composition in United States populations
Source: Front Plant Sci. 2024 Oct 24;15:1380624. doi: 10.3389/fpls.2024.1380624 (PMC11540636; doi:10.3389/fpls.2024.1380624)
Supplement: Supplementary file 1 [file Table1.docx]

Supplementary Material

**Supplementary Table S1.** Collection time and location data for all moths collected and genotyped from 2022-2023. Collection events are separated by nightly capture data. Trap type describes manual traps, which were traditional bucket style traps that were set out in the “early” and “late” portion of the evening and automated traps that generated hourly trap capture data across the evening. N = total number of C-strain and R-strain samples collected at each location and date. Temporal data was determined based on the time of collection (manual) or through the analysis of hourly images generated by the automated traps (automatic).

| Location | GPS Coordinates | Date | Host Plant | Trap Type | Sampling Event | Strain | Proportion Early | Proportion Late | N |
| --- | --- | --- | --- | --- | --- | --- | --- | --- | --- |
| Belle Glade, Florida | 26.6532, – 80.6389 | April 2022 | Sorghum/Maize | Manual | April 5, 2022 | C | 0.91 | 0.09 | 22 |
|  |  |  |  |  |  | R | 0 | 1 | 15 |
|  |  |  |  |  | April 6-8, 2022 | C | 1 | 0 | 18 |
|  |  |  |  |  |  | R | 0 | 1 | 19 |
|  |  | July 2023 | Rice/Sod | Manual | July 26, 2023 | C | 1 | 0 | 1 |
|  |  |  |  |  |  | R | 0 | 1 | 10 |
|  |  |  |  |  | July 27, 2023 | C | 1 | 0 | 3 |
|  |  |  |  |  |  | R | 0 | 1 | 10 |
|  |  |  |  |  | July 28, 2023 | C | 1 | 0 | 1 |
|  |  |  |  |  |  | R | 0 | 1 | 10 |
|  |  | Sept. 2023 | Sorghum/Maize | Auto | September 14, 2023 | C | 0 | 0 | 0 |
|  |  |  |  |  |  | R | 0 | 1 | 3 |
|  |  |  |  |  | September 15, 2023 | C | 0 | 1 | 3 |
|  |  |  |  |  |  | R | 0 | 1 | 18 |
|  |  |  |  |  | September 16, 2023 | C | 0.50 | 0.50 | 2 |
|  |  |  |  |  |  | R | 0.06 | 0.94 | 16 |
|  |  |  |  |  | September 17, 2023 | C | 0.33 | 0.67 | 12 |
|  |  |  |  |  |  | R | 0.05 | 0.95 | 22 |
| College Station, Texas | 30.5495, – 96.4367 | June 2023 | Sorghum | Auto | May 30, 2023 | C | 0 | 1 | 1 |
|  |  |  |  |  |  | R | 0 | 0 | 0 |
|  |  |  |  |  | June 11, 2023 | C | 1 | 0 | 1 |
|  |  |  |  |  |  | R | 0 | 0 | 0 |
|  |  |  |  |  | June 12, 2023 | C | 1 | 0 | 1 |
|  |  |  |  |  |  | R | 0 | 0 | 0 |
|  |  |  |  |  | June 13, 2023 | C | 1 | 0 | 1 |
|  |  |  |  |  |  | R | 0 | 0 | 0 |
|  |  |  |  |  | June 15, 2023 | C | 1 | 0 | 4 |
|  |  |  |  |  |  | R | 0 | 0 | 0 |
|  |  |  |  |  | June 18, 2023 | C | 0 | 1 | 1 |
|  |  |  |  |  |  | R | 0 | 0 | 0 |
|  |  |  |  |  | June 21, 2023 | C | 0.56 | 0.44 | 9 |
|  |  |  |  |  |  | R | 0 | 0 | 0 |
|  |  |  |  |  | June 22, 2023 | C | 0 | 1 | 4 |
|  |  |  |  |  |  | R | 0 | 0 | 0 |
|  |  |  |  |  | June 23, 2023 | C | 0.67 | 0.33 | 18 |
|  |  |  |  |  |  | R | 0 | 0 | 0 |
|  |  |  |  |  | June 24, 2023 | C | 0.75 | 0.25 | 4 |
|  |  |  |  |  |  | R | 0 | 0 | 0 |
|  |  |  |  |  | June 25, 2023 | C | 0 | 1 | 2 |
|  |  |  |  |  |  | R | 0 | 0 | 0 |
|  |  | July 2023 | Sorghum | Auto | July 11, 2023 | C | 0.64 | 0.36 | 14 |
|  |  |  |  |  |  | R | 0 | 0 | 0 |
|  |  |  |  |  | July 12, 2023 | C | 0.8 | 0.2 | 20 |
|  |  |  |  |  |  | R | 0 | 0 | 0 |
|  |  |  |  |  | July 13, 2023 | C | 0.25 | 0.75 | 4 |
|  |  |  |  |  |  | R | 0 | 0 | 0 |
|  |  | Oct. 2023 | Sorghum | Auto | October 15, 2023 | C | 1 | 0 | 1 |
|  |  |  |  |  |  | R | 0 | 0 | 0 |
|  |  |  |  |  | October 17, 2023 | C | 1 | 0 | 1 |
|  |  |  |  |  |  | R | 0 | 0 | 0 |
|  |  |  |  |  | October 18, 2023 | C | 1 | 0 | 2 |
|  |  |  |  |  |  | R | 0 | 0 | 0 |
|  |  |  |  |  | October 20, 2023 | C | 0 | 1 | 1 |
|  |  |  |  |  |  | R | 0 | 0 | 0 |
|  |  |  |  |  | October 22, 2023 | C | 1 | 0 | 4 |
|  |  |  |  |  |  | R | 0 | 0 | 0 |
|  |  |  |  |  | October 23, 2023 | C | 0.50 | 0.50 | 2 |
|  |  |  |  |  |  | R | 0 | 0 | 0 |
|  |  |  |  |  | October 24, 2023 | C | 0.33 | 0.67 | 3 |
|  |  |  |  |  |  | R | 0 | 0 | 0 |
|  |  |  |  |  | October 25, 2023 | C | 0.75 | 0.25 | 8 |
|  |  |  |  |  |  | R | 0 | 0 | 0 |
|  |  |  |  |  | October 27, 2023 | C | 1 | 0 | 1 |
|  |  |  |  |  |  | R | 0 | 0 | 0 |
|  |  |  |  |  | October 29, 2023 | C | 0 | 1 | 1 |
|  |  |  |  |  |  | R | 0 | 0 | 0 |
|  |  | Nov 2023 | Sorghum | Auto | November 3, 2023 | C | 1 | 0 | 3 |
|  |  |  |  |  |  | R | 1 | 0 | 3 |
|  |  |  |  |  | November 4, 2023 | C | 1 | 0 | 1 |
|  |  |  |  |  |  | R | 0 | 0 | 0 |
|  |  |  |  |  | November 5, 2023 | C | 0 | 0 | 0 |
|  |  |  |  |  |  | R | 0 | 1 | 2 |
|  |  |  |  |  | November 6, 2023 | C | 0 | 1 | 1 |
|  |  |  |  |  |  | R | 0 | 1 | 1 |
|  |  |  |  |  | November 7, 2023 | C | 0 | 1 | 1 |
|  |  |  |  |  |  | R | 0 | 0 | 0 |
|  |  |  |  |  | November 8, 2023 | C | 0 | 0 | 0 |
|  |  |  |  |  |  | R | 0 | 1 | 1 |
|  |  |  |  |  | November 11, 2023 | C | 0 | 0 | 0 |
|  |  |  |  |  |  | R | 0.2 | 0.8 | 5 |
|  |  |  |  |  | November 13, 2023 | C | 0 | 0 | 0 |
|  |  |  |  |  |  | R | 0.50 | 0.50 | 16 |
|  |  |  |  |  | November 14, 2023 | C | 0 | 0 | 0 |
|  |  |  |  |  |  | R | 0.33 | 0.67 | 3 |
